# Bacteroides fragilis Strain ZY-312 Defense against Cronobacter sakazakii-Induced Necrotizing Enterocolitis *In Vitro* and in a Neonatal Rat Model

**DOI:** 10.1128/mSystems.00305-19

**Published:** 2019-08-06

**Authors:** Hongying Fan, Zhenhui Chen, Ruqin Lin, Yangyang Liu, Xianbo Wu, Santhosh Puthiyakunnon, Ye Wang, Bo Zhu, Qiwei Zhang, Yang Bai, Fachao Zhi

**Affiliations:** a Department of Microbiology, Guangdong Provincial Key Laboratory of Tropical Disease Research, School of Public Health, Southern Medical University, Guangzhou, China; b Guangdong Provincial Key Laboratory of Gastroenterology, Institute of Gastroenterology of Guangdong Province, Department of Gastroenterology, Nanfang Hospital, Southern Medical University, Guangzhou, China; c Guangzhou ZhiYi Biotechnology Co., Ltd., Guangzhou, China; Vall d'Hebron Research Institute

**Keywords:** apoptosis, *Bacteroides fragilis*, *Cronobacter sakazakii*, inflammasomes, necrotizing enterocolitis, pyroptosis

## Abstract

Cronobacter sakazakii is an important pathogen associated with the development of necrotizing enterocolitis (NEC), infant sepsis, and meningitis. Several randomized prospective clinical trials demonstrated that oral probiotics could decrease the incidence of NEC. Previously, we isolated and characterized a novel probiotic, Bacteroides fragilis strain ZY-312. However, it remains unclear how ZY-312 protects the host from the effects of C. sakazakii infection. To understand the underlying mechanisms triggering the probiotic effects, we tested the hypothesis that there was cross talk between probiotics/probiotics-modulated microbiota and the local immune system, governed by the permeability of the intestinal mucosa, using *in vitro* and *in vivo* models for the intestinal permeability. The probiotic effects of ZY-312 on intestinal epithelial cells were first examined, and the results revealed that ZY-312 inhibited C. sakazakii invasion, C. sakazakii-induced dual cell death (pyroptosis and apoptosis), and epithelial barrier dysfunction *in vitro* and *in vivo*. The presence of ZY-312 also resulted in decreased expression of an inflammasome (NOD-like receptor family member pyrin domain-containing protein 3 [NLRP3]), caspase-3, and serine protease caspase-1 in a neonatal rat model. Furthermore, ZY-312 significantly modulated the compositions of the intestinal bacterial communities and decreased the relative abundances of *Proteobacteria* and *Gammaproteobacteria* but increased the relative abundances of *Bacteroides* and *Bacillus* in neonatal rats. In conclusion, our findings have shown for the first time that the probiotic B. fragilis ZY-312 suppresses C. sakazakii-induced NEC by modulating the proinflammatory response and dual cell death (apoptosis and pyroptosis).

**IMPORTANCE** Cronobacter sakazakii is an opportunistic pathogenic bacterium that can cause necrotizing enterocolitis (NEC). However, the mechanism of pathogenicity of C. sakazakii is largely unknown. Here we have now demonstrated that apoptotic and pyroptotic stimuli are effectors of C. sakazakii-induced NEC. Previously, we isolated a novel probiotic strain candidate from fecal samples from healthy infants and characterized it as Bacteroides fragilis strain ZY-312. Functional characterization reveals that ZY-312 inhibited C. sakazakii invasion, restoring epithelial barrier dysfunction, decreasing the expression of inflammatory cytokines, and reducing dual cell death (pyroptosis and apoptosis). Furthermore, the presence of ZY-132 was sufficient to hinder the adverse reaction seen with C. sakazakii in a C. sakazakii-induced NEC model. Taking the results together, our study demonstrated the utility of ZY-312 as a promising probiotic agent for the prevention of NEC.

## Abstract (precis)

Cronobacter sakazakii is an opportunistic pathogenic bacterium that can cause necrotizing enterocolitis (NEC). However, the mechanism of pathogenicity of C. sakazakii is largely unknown. Here we have now demonstrated that apoptotic and pyroptotic stimuli are effectors of C. sakazakii-induced NEC. Previously, we isolated a novel probiotic strain candidate from fecal samples from healthy infants and characterized it as Bacteroides fragilis strain ZY-312. Functional characterization reveals that ZY-312 inhibited C. sakazakii invasion, restoring epithelial barrier dysfunction, decreasing the expression of inflammatory cytokines, and reducing dual cell death (pyroptosis and apoptosis). Furthermore, the presence of ZY-132 was sufficient to hinder the adverse reaction seen with C. sakazakii in a C. sakazakii-induced NEC model. Taking the results together, our study demonstrated the utility of ZY-312 as a promising probiotic agent for the prevention of NEC.

## INTRODUCTION

Necrotizing enterocolitis (NEC) is a severe intestinal inflammation that affects approximately 20% of preterm neonates and is the leading cause of death and long-term disability from gastrointestinal (GI) diseases among preterm infants (1). Rates of mortality (20% to 40%) and morbidity, including long-term neuronal developmental disorders, are persistently high, especially in infants with very low birth weight (2). NEC is a serious clinical condition affecting neonates, and its treatment remains a pharmacological challenge. Risk factors associated with the development of NEC include premature birth, enteral feeding, altered enteric mucosal integrity, and the presence of pathogenic organisms (3, 4). Various opportunistic pathogens are also believed to play important roles in the pathogenesis of NEC. One such organism is Cronobacter sakazakii, an emerging opportunistic pathogen, which has been isolated in hospital laboratories in association with several NEC outbreaks (5). C. sakazakii is a Gram-negative, rod-shaped, and non-spore-forming pathogen in the Enterobacteriaceae family. It is commonly found in dairy products, and contamination of infant formula with C. sakazakii leads to NEC outbreak (6). C. sakazakii has been hypothesized to be associated with the development of NEC, because oral introduction of C. sakazakii into experimental NEC models has been shown previously to exacerbate intestinal injuries (5, 7, 8). As a result, C. sakazakii is used as a model microorganism for the study of opportunistic bacteria in the pathogenesis of NEC. A previous systematic review and meta-analysis of such studies found that probiotics significantly reduced the severity of NEC by affecting immunity, inflammation, tissue injury, gut barrier, and intestinal dysbiosis (9). A recent study also demonstrated that administration of multiple-strain probiotics was a feasible and effective strategy to prevent the pathogenesis of NEC and to prevent NEC-related mortality (10, 11). However, there have been no standardized clinical studies to assess either the most effective probiotic bacterial species or the dosages required to combat the pathogens responsible for the development of NEC.

Previous studies have shown that commensal and probiotic bacteria regulate the intestinal defense system (including barrier function, mucin, and secretory IgA), inflammation, and homeostatic processes, such as cell proliferation and apoptosis (12–14). Immature intestinal host defenses play a critical role in the pathogenesis of neonatal intestinal inflammatory diseases such as NEC, and commensal bacteria are involved in the promotion of the maturation of these host defenses (15–17). The specific host defenses promoted by commensal and probiotic bacteria include intestinal epithelial cell proliferation and apoptosis, innate immune regulation, and epithelial barrier function (18, 19). Bacteroides fragilis, a commensal species and an anaerobic Gram-negative bacterium found in the human gut, may be involved in the control of the pathogenesis of neonatal intestinal inflammatory diseases (20). Recently, nontoxigenic B. fragilis was shown to have beneficial effects on host health by promoting immune system maturation, suppressing abnormal inflammation, and altering the structure of the intestinal microflora (21–23). Another study also showed that B. fragilis improved autism symptoms in mouse models, even in the absence of polysaccharide A (PSA) (24). B. fragilis has, therefore, been suggested as a candidate probiotic with the ability to inhibit pathogenic bacteria (25). Several theoretical mechanisms have been proposed to explain the effects of probiotic bacteria on pathogens, including competition for binding sites on the intestinal wall for nutrients, the production of antibacterial compounds and lactic acid, and indirect effects via immunomodulation (26–28). To investigate the potentials and the mechanisms of action of probiotics, we previously isolated a novel B. fragilis strain (ZY-312). Our study showed that ZY-312 inhibits the growth of Vibrio parahaemolyticus, an anaerobic gut pathogen that causes acute gastroenteritis. ZY-312 also shortened the duration of V. parahaemolyticus colonization in the intestines of mice and reduced the cellular damage caused by the pathogen (25). Programmed cell death (PCD) is a crucial process that occurs in response to microbial infections, and there is a complex interplay between different pathways in the innate immune system (29, 30). Apoptosis and pyroptosis are two pathways of PCD that are associated with bacterial infections, with substantially different outcomes. Apoptosis was demonstrated in C. sakazakii-induced host immune responses (5). However, it was not clear whether pyroptosis plays a role in C. sakazakii-induced inflammatory response. Pyroptotic cell death is triggered by caspase-1 activation via various inflammasomes and results in lysis of the affected cell (31). The inflammasome, a multimeric protein complex consisting of Nod-like receptors (NLRs), ASC (Apoptosis-associated speck-like protein containing a CARD), and caspase-1, was shown to be important in the maturation and secretion of interleukin-1β (IL-1β) and its related family members. In particular, the NLRP3 inflammasome has been extensively studied and can be activated by a variety of stimuli, including microbial infection (12–14). The NLRP3 inﬂammasome could be a suitable drug target for the treatment of infectious diseases, such as NEC, which potentially prevents the risk of developing antibiotic resistance (21).

Here, we hypothesized that there is cross talk between B. fragilis strain ZY-312 and bacteria of the microbiota modulated by ZY-312 and that the local gut immune system plays a crucial role in ZY-312-mediated modulation of PCD and inflammatory responses to NEC. To test this hypothesis, the effects of ZY-312 on the pathogenicity of C. sakazakii were examined using *in vitro* and *in vivo* animal models.

## RESULTS

### ZY-312 inhibited C. sakazakii adherence to intestinal cells through modulation of MUC2 expression in C. sakazakii-infected cells.

**FIG 1:**
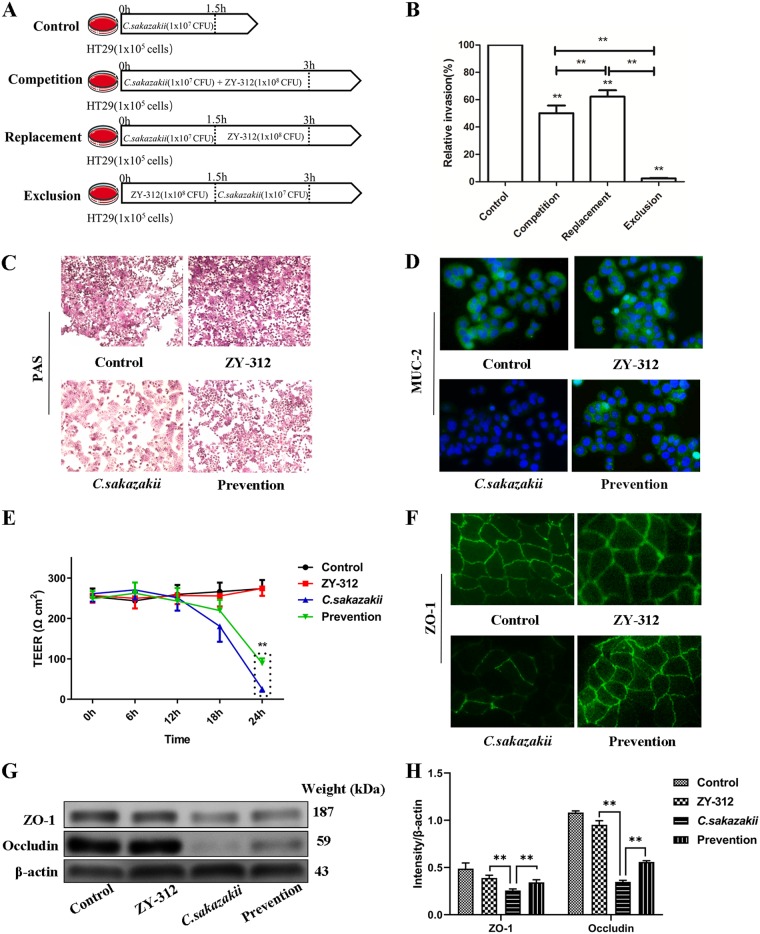
ZY-312 inhibits C. sakazakii invasion of HT-29 cells, modulates mucous glycoprotein production and MUC2 expression, and attenuates C. sakazakii-induced decreases in levels of Caco-2 monolayer TEER and expression of tight junction protein. (A) Protocol of invasion assay. (B) The effects of ZY-312 on C. sakazakii invasion of HT-29 cells. Data are expressed as relative levels of invasion compared with the control (100%) without ZY-312 pretreatment and are shown as percentages ± SD (*n* = 9). (C) HT-29 cells were treated as indicated and stained with PAS to reveal mucin production. (D) HT-29 cells were immune stained with anti-MUC2 antibody (in green) and FITC-conjugated secondary antibody and examined by fluorescence microscopy before imaging. The nuclei were counterstained using DAPI (in blue). (E) TEER values for Caco-2 monolayers treated and infected with C. sakazakii (MOI = 100) for 6 h, 12 h, 18 h, and 24 h. (F) Caco-2 monolayers were fluorescently stained to highlight the tight junction protein ZO-1 (in green) and imaged using a fluorescence microscope. (G) Representative Western blot showing ZO-1 and occludin (OCLN) expression in Caco-2 whole-cell protein extracts. β-Actin was used as an indicator of protein loading. (H) Data represent results from three independent experiments. **, *P < *0.01.

Both competition and replacement assays showed that ZY-312 expelled C. sakazakii from HT-29 cells (*P < *0.001) with almost 95% efficiency (*P < *0.01), demonstrating that ZY-312 had a preventive effect on C. sakazakii infection in *in vitro* cell models (Fig. 1B).

The effects of ZY-312 pretreatment on the production of mucins and MUC2 in HT-29 cells were evaluated. Periodic acid-Schiff stain (PAS) results showed that C. sakazakii infection led to a general downregulation of mucin production and that ZY-312 pretreatment in HT-29 cells inhibited the C. sakazakii-induced decrease in mucin production (Fig. 1C). MUC2 levels were also directly assessed by immunostaining, which demonstrated that C. sakazakii disrupted MUC2 expression and that ZY-312 pretreatment was able reverse this effect in HT-29 cells (Fig. 1D).

### ZY-312 inhibited C. sakazakii-induced decrease in transepithelial electrical resistance (TEER) and the expression of tight junction proteins in Caco-2 monolayers.

We further examined whether ZY-312 or C. sakazakii influenced the gut barrier function and found that infection with C. sakazakii reduced the TEER of the Caco-2 monolayers in a time-dependent manner. In contrast, stable TEER values were observed in the ZY-312 group. When Caco-2 cells were preincubated with ZY-312 for 3 h before infection with C. sakazakii, the C. sakazakii-induced reduction of TEER was alleviated but still decreased by 18 h after infection (Fig. 1E). We also evaluated the effects of ZY-312 on epithelial barrier function in the intestine cell lines by detecting tight junction protein expression. Immunostaining showed that ZY-312 pretreatment attenuated the C. sakazakii-induced decrease in ZO-1 protein expression observed in the Caco-2 monolayers (Fig. 1F). Western blot analysis showed that C. sakazakii infection decreased the expression of ZO-1 and occludin (OCLN) proteins in Caco-2 cells, whereas the ZY-312 group showed no increase in protein expression. In Caco-2 cells pretreated with ZY-312, there was increased ZO-1 and occludin (OCLN) protein expression compared with cells infected with C. sakazakii only. In addition, the level of expression of ZO-1 but not occludin (OCLN) was higher in ZY-312-treated cells than in the prevention group (Fig. 1G and H).

### ZY-312 ameliorated the deleterious effects of C. sakazakii on intestinal integrity and attenuated clinical symptoms and intestinal inflammation during C. sakazakii-induced NEC in neonatal rats.

**FIG 2:**
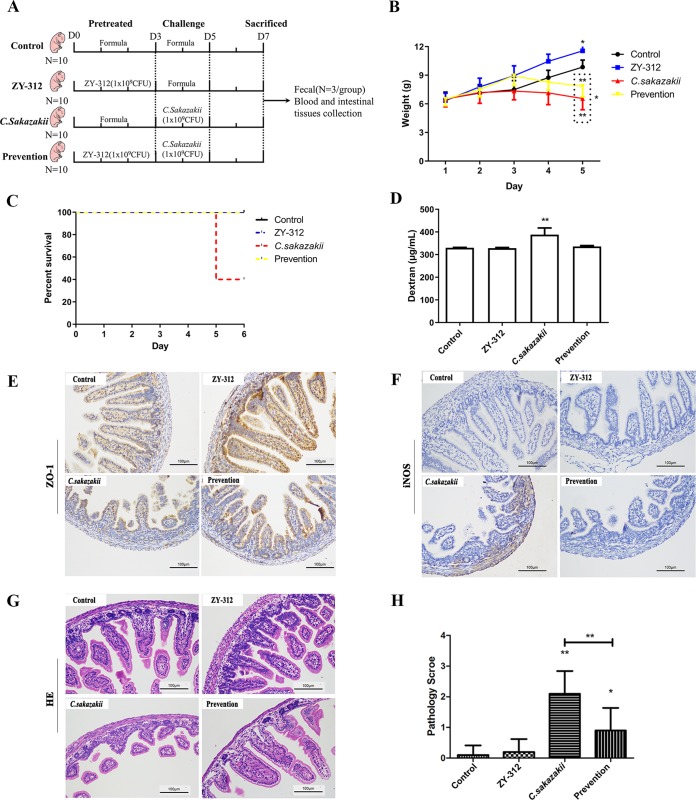
ZY-312 ameliorates C. sakazakii-induced effects on the intestinal integrity and attenuates clinical symptoms and intestinal inflammation during the pathological process of C. sakazakii-induced NEC in neonatal rats. (A) Experimental design for four groups. Rats were fed with 0.2 ml of a formula containing 1 × 10^9^ CFU of ZY-312 or formula alone once per day from day 0 to day 3. Then, the rats were fed with a 0.2 ml C. sakazakii formula (1 × 10^9^ CFU) once per day and exposed thrice to a hypoxia exposure regime (5% O_2_ and 95% N_2_) or to formula alone from day 3 to day 5. On day 7, rats were sacrificed and feces samples (*n* = 3 in each group), blood, and intestinal tissues were collected for the next experiments. (B) Body weight changes of neonatal rats during C. sakazakii infection. (C) Mortality rate evaluation in neonatal rats during C. sakazakii infection. (D) The intestinal barrier permeability was determined by quantifying serum concentrations of FITC-dextran. (E) Immunohistochemical staining showing the expression levels of the tight junction protein ZO-1 in the intestines of neonatal rats. (F) Immunostaining of iNOS in the intestinal tissue of neonatal rats. (G) Representative images of intestinal tissue (stained with H&E) from neonatal rats subjected to various treatments. (H) Semiquantitative pathology scores of intestinal tissues from neonatal rats subjected to various treatments. *, *P < *0.05; **, *P < *0.01.

To evaluate the effects of C. sakazakii on intestinal barrier function *in vivo*, after the challenge test with the C. sakazakii strain, the rats showed clinical manifestations such as weight loss, loss of appetite, abdominal flatulence, and subsequent death. C. sakazakii infection often results in NEC; however, this condition can be ameliorated by probiotic pretreatment. In this study, C. sakazakii infection led to severe weight loss whereas ZY-312 treatment promoted weight gain. Pretreatment with ZY-312 before C. sakazakii infection was effective in reducing weight loss. Monitoring changes in the body weight of the rats, the C. sakazakii group and the prevention group showed a decrease in body weight after day 3, and on day 5, the ZY-312 group body weight was higher than the phosphate-buffered saline (PBS) group weight (*P < *0.05), while the C. sakazakii group weight was lower than that of the prevention group (*P*< 0.001) (Fig. 2B). Evaluating the mortality of the rats, we found that the C. sakazakii model group rats began to die on the fifth day (the mortality rate reached 60%) and that no deaths were found in the other three groups (Fig. 2C). Therefore, these results indicated that ZY-312 treatment was able to improve the survival rate of the rats and prevent C. sakazakii infections.

We assessed the gut permeability and the level of expression of the tight junction protein ZO-1 in neonatal rats. C. sakazakii infection alone resulted in increased intestinal permeability to a 4-kDa macromolecular fluorescent probe. Our data showed that the neonates that were pretreated with ZY-312 before C. sakazakii infection had lower serum levels of fluorescein isothiocyanate (FITC)-dextran than the C. sakazakii-infected neonates (Fig. 2D). This indicated that ZY-312 prevented C. sakazakii-induced intestinal barrier injury and contributed to the maintenance of the mucosal barrier integrity. Further immunohistochemical staining showed that the ZY-312-treated group had increased expression of ZO-1 protein in the ileum compared with the control group. These data also confirmed that C. sakazakii decreased ZO-1 protein expression in the neonates and that ZY-312 pretreatment attenuated this downregulation (Fig. 2E).

We also noticed that C. sakazakii infection stimulated inducible nitric oxide synthase (iNOS) production and that ZY-312 pretreatment suppressed this effect (Fig. 2F). Furthermore, histological examination of intestinal segments from C. sakazakii-infected animals revealed a significant loss of villi and infiltration of neutrophils. Exposure to ZY-312 alone had no effect on intestinal integrity, similarly to what was observed in the control animals. Treatment with ZY-312 prior to infection with C. sakazakii decreased the occurrence and extent of intestinal injury (Fig. 2G and H).

### Pretreatment with ZY-312 diminished the expression of proinflammatory cytokines and promoted anti-inflammatory factors in neonatal rats.

**FIG 3:**
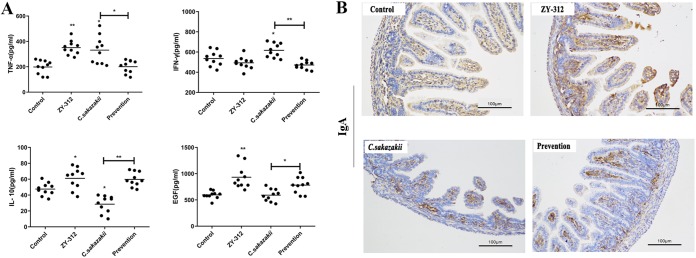
Pretreatment with ZY-312 reduces the expression of proinflammatory cytokines and promotes anti-inflammatory factors in neonatal rats. (A) Serum levels of TNF, IFN-γ, IL-10, and EGF in neonatal rats. *, *P < *0.05; **, *P < *0.01. (B) Immunostaining of IgA in the intestinal tissue of neonatal rats.

To delineate the relationship between ZY-312 administration and the modulation of either proinflammatory or anti-inflammatory factors, an enzyme-linked immunosorbent assay (ELISA) was used to detect the expression of helper T-cell 1 (TH1) cytokines (tumor necrosis factor [TNF] and gamma interferon [IFN-γ]), helper T-cell 2 (TH2), cytokine (IL-10), and epidermal growth factor (EGF). These data showed that C. sakazakii induced increased expression of TNF and IFN-γ and that the increase was prevented by pretreatment with ZY-312. In addition, a C. sakazakii-induced IL-10 expression decrease was prevented by pretreatment with ZY-312 (Fig. 3A). We also tested whether ZY-312 promoted the expression of EGF in rats after C. sakazakii infection. EGF receptor inactivation in rats can lead to intestinal lesions resembling NEC, and rats fed with EGF-supplemented rat milk displayed a reduced incidence of NEC and a lower level of disease severity (32). Our data showed that serum EGF levels were increased in the ZY-312 group and that there was no decrease in serum EGF levels in the C. sakazakii group. However, EGF levels in the group pretreated with ZY-312 were higher than in the group exposed to C. sakazakii alone (Fig. 3A). These results indicated that ZY-312 might enhance the expression of EGF and IL-10 and thus might prevent the development of C. sakazakii-induced NEC.

We also compared the levels of production of IgA in the intestines of rats by the use of immunohistochemical staining (Fig. 3B). We found that IgA expression was higher in the ZY-312 group than in the control group. In contrast, IgA levels in the C. sakazakii-only group were lower, and the group pretreated with ZY-312 had slightly increased IgA levels.

### ZY-312 inhibited C. sakazakii-induced programmed cell death (PCD).

**FIG 4:**
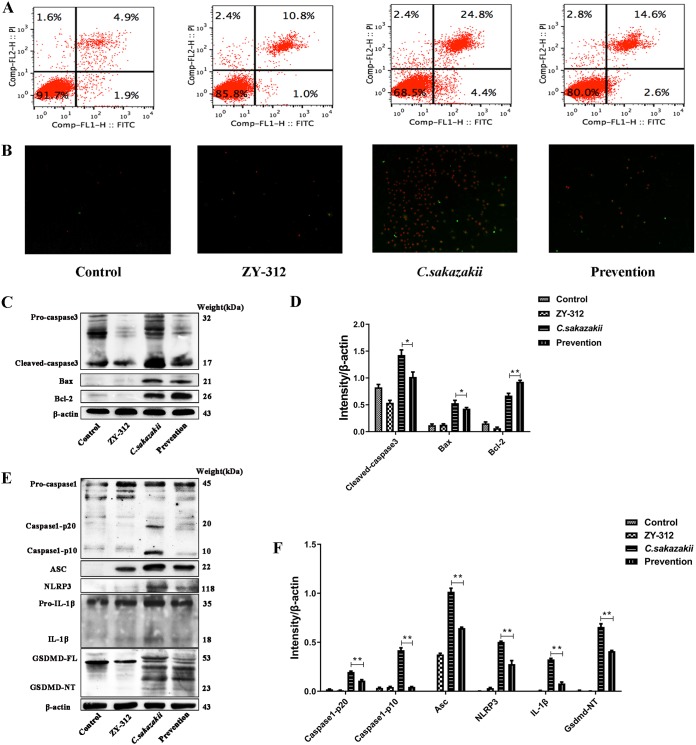
ZY-312 reduces C. sakazakii-induced pyroptosis and apoptosis. (A and B) C. sakazakii-induced programmed cell death in HT-29 was ameliorated by pretreatment with ZY-312. Flow cytometry (A) and fluorescence microscope (B) were used to examine the results of staining of FITC and PI. (C) Western blot analysis shows that ZY-312 suppressed C. sakazakii-induced NEC by modulating apoptosis through caspase-3, Bax, and Bcl-2. β-Actin was used as an indicator of protein loading. (D) Data represent results from three independent experiments. (E) Western blot analysis shows that ZY-312 suppressed C. sakazakii-induced NEC by modulating pyroptosis through the NLRP3 inflammasome (caspase-1, ASC, NLRP3), IL-1β, and Gsdmd. β-Actin was used as an indicator of protein loading. (F) Data represent results from three independent experiments. *, *P < *0.05; **, *P < *0.01.

As indicated in Fig. 4A (upper panel), C. sakazakii-infected cells showed a high degree of programmed cell death (29.2%). Although ZY-312 exposure led to a small increase in programmed cell death in HT-29 cells (11.8%) compared with control cells (6.8%), ZY-312 pretreatment inhibited C. sakazakii-induced programmed cell death (17.2%). Those results were consistent with the figures captured by fluorescence microscope (Fig. 4B). However, it is unknown whether the cell death was induced by apoptosis or pyroptosis.

The apoptosis-related proteins included caspase-3, caspase-6, and the Bcl-2 family. Bcl-2 expression and Bax expression can activate caspase-3 and p53 and further induce apoptosis. Western blotting revealed that ZY-312 had no effect on the expression of Bcl-2 and Bax compared with their levels of expression in controls. As shown in Fig. 4C, we found that the cleaved caspase3 and Bax protein levels in the prevention group were lower than those in the C. sakazakii group. In contrast, the Bcl-2 protein level was higher in the group pretreated with ZY-312 than in the C. sakazakii group. Therefore, ZY-312 could suppress C. sakazakii-induced NEC by modulating apoptosis (Fig. 4C and D).

To examine whether ZY-312 could inhibit C. sakazakii-induced inflammatory responses through the modulation of the NLRP3 inﬂammasome pathway, we determined the levels of expression and activation of the NLRP3 inflammasome along with pyroptosis-related proteins using Western blotting. The results showed that the expression levels of the NLRP3 inflammasome and ASC increased in the C. sakazakii group and that the levels of activation of NLRP3, caspase-1 p20, caspase-1 p10, IL-1β, and gasdermin D (GSDMD) were significantly increased in the C. sakazakii group. These observed results were reversed by ZY-312 treatment (Fig. 4E and F). It is worth mentioning that ZY-312 was able to increase the expression of only ASC compared with the control group, whereas C. sakazakii could elevate the levels of expression of all of the inflammatory mediators of the NLRP3 inflammasome pathway along with pyroptosis-related proteins. ZY-312 could suppress C. sakazakii-induced NEC by modulating proinflammatory responses and pyroptosis.

### ZY-312 affected the compositions of the intestinal bacterial communities in neonatal rats.

**FIG 5:**
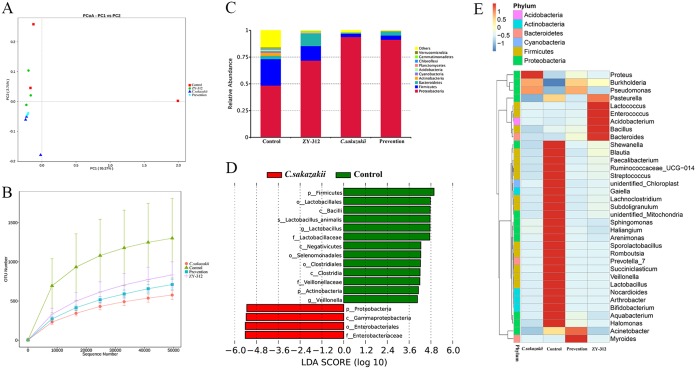
ZY-312 affects the intestinal bacterial communities in neonatal rats. The compositions of intestinal bacterial communities in neonatal rats were investigated using Illumina sequencing of the 16S rRNA gene (*n* = 3 in each group). (A) PCoA data based on OTUs in all groups. (B) Rarefaction curve showing the relative abundances of OTUs in all groups. (C) Relative abundances of the most abundant bacterial phyla in different groups. (D) Significantly different bacterial biomarkers, determined by the least discriminant analysis (LDA) effect size, were identified in C. sakazakii relative to the control group. (E) A heat map constructed with the top 35 most abundant genera.

Finally, we evaluated the effects of ZY-312 and C. sakazakii on the composition of the intestinal microbiota of rats (*n* = 3 in each group) using Illumina sequencing of the 16S rRNA V4 region. Principal-coordinate analysis (PCoA) demonstrated that our samples were acceptable in each group. However, in the control group and the ZY-312 group, interindividual variability was observed (Fig. 5A). Rarefaction curves showed the relative abundances of the operational taxonomic units (OTUs) in all groups (Fig. 5B). In the case of C. sakazakii, the numbers of OTUs were dramatically decreased and this change was able to be rescued in the prevention group. Also, the relative abundances of three representative species, *Proteobacteria*, *Firmicutes*, and *Bacteroidetes*, were detected and are shown, although there was a decrease in the abundance of *Bacteroidetes* in C. sakazakii-infected neonates. The level of the decrease was lower in the ZY-312 treatment and ZY-312 pretreatment groups (Fig. 5C). The genus *Proteus* were more abundant in the C. sakazakii-treated group, while the *Bacteroides*, *Bacillus*, *Acidobacterium*, *Enterococcus*, *Lactococcus*, and *Pasteurella* genera were more abundant in the ZY-312-treated group. *Myroides* and Acinetobacter were more common in the group pretreated with ZY-312 before C. sakazakii infection. Furthermore, *Proteobacteria* showed reduced relative abundance and *Bacteroidetes* showed decreased relative abundance in the prevention group (Fig. 5D). The linear discriminant analysis effect size (LEfSe) algorithm was used to search statistically significantly different bacterial groups between the C. sakazakii and control rats. It was found that the relative abundances of *Proteobacteria*, *Gammaproteobacteria*, *Enterobacteriales*, and Enterobacteriaceae in the C. sakazakii group were significantly higher than in the control group (Fig. 5E). Therefore, ZY-312 can prevent C. sakazakii-induced NEC by regulating the intestinal flora.

## DISCUSSION

NEC, one of the most serious of the medical conditions that affect neonates, has a poor treatment outcome. Evidence suggests that the interaction between indigenous bacteria and the intestine of neonates plays a crucial role in the pathogenesis of NEC (1). The potential mechanisms include an increased barrier to bacterial pathogens and their products, modification of host response to microbial agents, augmentation of GI mucosal responses, enhancement of enteral nutrition, and upregulation of immune responses (33). Clinical trial data suggested that there was a significant reduction in the risk of NEC development and in NEC mortality after probiotic supplementation in preterm neonates with very low birth weight (VLBW) compared with controls (2, 33–35). However, as indicated in numerous studies, there is still a considerable variation in the treatment modality for neonates with sepsis and NEC (2, 33–35). These variations include type, dose, duration of probiotic supplementation, age of commencement, and antibiotics use. The optimum types of probiotic supplements remain to be determined. Here, our study showed that treatment with the potential probiotic B. fragilis ZY-312 improved intestinal epithelial barrier function through inhibition of invasion, programmed cell death, and modulation of MUC2 and tight junction protein expression *in vitro*. In addition, ZY-312 treatment attenuated C. sakazakii-induced intestinal inflammation, epithelial barrier damage, and microbiota disruption *in vivo* in the intestines of neonatal rats.

There are two subsequent steps essential for pathogen entry into host cells: bacterial adhesion and invasion. C. sakazakii binding and invasion of intestinal epithelial cells are required for the bacterium to cross the gut barrier *in vivo* (36). Inhibiting pathogen adhesion to epithelial cells may therefore prevent opportunistic infection. In competition and replacement assays, it was shown that ZY-312 expelled C. sakazakii from HT-29 cells (*P* < 0.001) with almost 95% efficiency (*P* < 0.01). The results have demonstrated that ZY-312 has the ability to inhibit C. sakazakii invasion of intestinal cells, which is consistent with the previous studies (37). The mucus layer (primarily composed of MUC2) that covers the surface of the intestinal epithelium plays an important role in protecting the integrity of the intestinal epithelial barrier. We found that ZY-312 attenuated C. sakazakii-induced disruption of MUC2 expression and mucus production *in vivo*, suggesting that ZY-312 improves mucus layer function by modulating MUC2 expression in the intestine.

Other important components of intestinal barrier integrity are tight junctions. Both ZO-1 and occludin (OCLN) are important tight junction proteins expressed by intestinal epithelial cells. Growing evidence has indicated that an increase in the permeability of the intestinal barrier precedes the development of NEC (38). Consistent with this, we found that ZY-312 pretreatment prevented membrane disruption induced by C. sakazakii. A decrease in the levels of expression of ZO-1 and occludin (OCLN) caused by C. sakazakii was reversed by pretreatment with ZY-312. We also found similar effects in neonatal rat models. In this study, we used immunohistochemical analysis of ZO-1 as a marker for membrane integrity to evaluate the changes in gut permeability. Although ZY-312 treatment alone *in vitro* did not affect the levels of protein expression of ZO-1 and occludin (OCLN) or enhance tight junction integrity, the results of this *in vivo* study performed with rat models showed that ZO-1 expression was enhanced in the ZY-312 group compared with the control group. We, therefore, hypothesize that ZY-312 acts on tight junctions and improves epithelial barrier function in rat models.

C. sakazakii species are known to induce apoptosis and immune responses (5). In this study, we have shown for the first time that C. sakazakii not only induces apoptosis but also elicits pyroptosis, triggered by caspase-1 activation via inflammasomes (39). The premature human gut is notable for displaying increased expression of various proinflammatory cytokines. Increasing evidence indicates that the dynamics in immune cells in NEC are actually attributable to the development of NEC, because the imbalances can favor a proinflammatory state (40). In this study, we found that C. sakazakii induced increased expression of proinflammatory markers tumor necrosis factor TNF and gamma interferon IFN-γ and decreased expression of anti-inflammatory marker IL-10. However, these effects could be modulated by pretreatment with ZY-312, suggesting that ZY-312 has the capability to regulate the immunologic balance between TH1 and TH2 responses. Furthermore, we also found that ZY-312 increased IgA and EGF levels in the intestines of rats. Taken together, these findings showed that ZY-312 effectively attenuated inflammation and epithelial barrier damage, thereby preventing the development of NEC.

Inflammatory responses are activated by inflammasomes (multiprotein oligomers), which are responsible for a more robust immune response to microbial pathogens and which promote the activation/maturation of inflammatory caspase-1 and proinflammatory cytokines such as IL-1β (39). Proteus mirabilis, through the NLRP3 inflammasome, can contribute to dextran sulfate sodium-induced colitis, indicating that disease outcomes are affected by the various commensal microbiota present in the intestine. A large number of studies on the pathogenic effects of C. sakazakii on NEC have focused only on apoptosis, a form of noninflammatory programmed cell death. In a murine model of C. sakazakii infection, we observed that both caspase-1 and caspase-3, responsible for the execution of pyroptosis and apoptosis, respectively, were significantly downregulated by ZY-312. C. sakazakii alone induced statistically significantly severe gut injury and animal death without ZY-312 treatment compared with C. sakazakii-infected animals treated with ZY-312. Therefore, ZY-312 may reduce levels of C. sakazakii-induced inflammatory programmed cell death (pyroptosis) by inhibiting caspase-1 and reducing IL-1β levels. This may be an important mechanism through which probiotics could be exploited to prevent inflammation.

The intestinal mucosal surface hosts about 70% of the cells of the entire immune system (6). It also serves as the home of a large number of microbial flora (microbiota). The establishment of a stable and diverse intestinal flora is a key requirement for optimal mucosal defense. Epithelial barrier damage and immune-mediated disorders are usually related to disruptions in the composition of the intestinal microbiota that occur during pathogen infection (41). Using Illumina sequencing of the 16S rRNA gene, we found that C. sakazakii infection led to a decreased number of OTUs and formation of a new, unique bacterial community, strongly indicating that C. sakazakii infection specifically changes the composition of the microbiota. Furthermore, pretreatment with ZY-312 rescue the decreased relative abundance and led to the preservation of *Bacteroides* abundance after C. sakazakii infection, suggesting that *Bacteroides* spp. contribute to the host resistance to bacterial pathogens. However, interindividual variability was observed in the control group and the ZY-312 group. It was reported previously that the gender difference of rats may influence the compositions of the intestinal bacterial communities (42, 43). Therefore, in this study, the gender difference of the rats and the limited number of only 3 rats in each group may explain this interindividual variability observation. Interestingly, we found that the samples were of higher (acceptable) quality in the ZY-312 group, the C. sakazakii group, and the prevention group than in the control group. It was reported previously that early exposure to a specific environment and a variety of microbial organisms can lead to lifelong colonization (44, 45). Therefore, we assumed that microbial organisms such as C. sakazakii and ZY-312 may have some effect on the convergence of intestinal bacterial communities in those groups. In conclusion, we propose a novel model of C. sakazakii-induced programmed cell death, with dual features of pyroptosis and apoptosis in intestinal epithelial cells. In particular, we have now unequivocally demonstrated that both apoptotic and pyroptotic stimuli contribute to the pathogenesis of C. sakazakii-caused NEC. In addition to these biological insights, this study demonstrated the utility of ZY-312 as a promising probiotic agent for the prevention and treatment of various intestinal diseases, including NEC.

**FIG 6:**
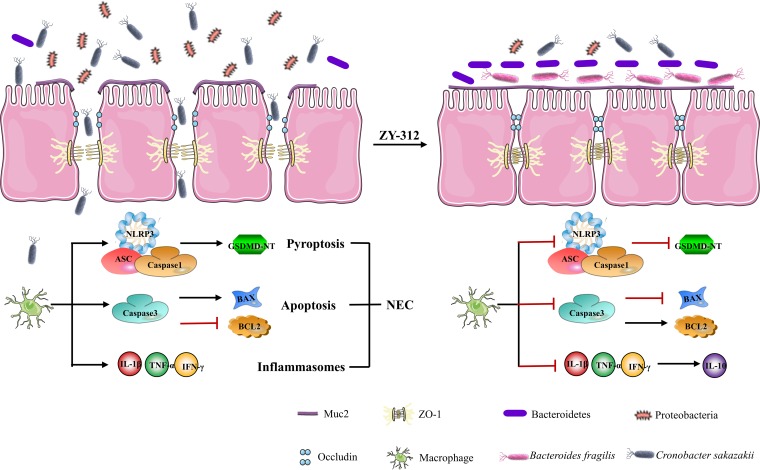
ZY-312 defenses against C. sakazakii-induced NEC.

In this study, we demonstrated for the first time that ZY-312 inhibited the deleterious effects of Cronobacter sakazakii infection through five independent processes (Fig. 6). First, ZY-312 may affect the compositions of the intestinal bacterial communities in C. sakazakii-induced NEC. Second, ZY-312 reversed the decreased expression of tight junction proteins (Muc2, occludin, and Zo-1) caused by C. sakazakii. Furthermore, C. sakazakii induced pyroptosis through NLRP3/caspase-1/IL-1β signaling. C. sakazakii activated three NLRP3 inflammasome related proteins (caspase-1, ASC and NLRP3). These observed results were reversed by ZY-312 treatment. Additionally, ZY-312 reversed the increased expression seen with caspase-3 and Bax and decreased expression of Bcl-2. That is, ZY-312 reversed the apoptosis induced by C. sakazakii. Finally, C. sakazakii increased expression of IL-1β, TNF, and IFN-γ, and those effects can be reversed by pretreatment with ZY-312.

Interestingly, we also found that the presence of B. fragilis strain ZY-312 alone had some effects on neonatal rats. The level of ASC in the ZY-312 group was increased compared with that in the control group. However, the levels of cleaved caspase-1 and of cell death shown by comparisons of those two groups remained the same. It was reported previously that a symbiosis factor (PSA) of B. fragilis signals through Toll-like receptor 2 (TLR2) directly on Foxp3(+) regulatory T cells to promote immunologic tolerance (22). TLR2 mediates interactions with the downstream MyD88 adapter molecule and therefore activates transcription factor NF-κB and recruits ASC (46). In the ZY-312 group, ZY-312 increased the level of TNF. It was also reported previously that B. fragilis increased the level of TNF-α when the bacteria invaded the abdominal cavity (47, 48). Upon elevation of the TNF-α levels, PSA was internalized by antigen-presenting cells (APCs) and, along with major histocompatibility complex class II (MHC-II), was presented to T cells to generate IL-10 for controlling excessive inflammation. Also, ZY-312 promoted a significant increase in the weight of animals. B. fragilis ZY-312 is a member of *Bacteroides* group. It was previously reported that the presence of Lactobacillus casei Zhang, also a member of *Bacteroides* group, was correlated with glucose tolerance and promoted glucose metabolism indirectly (49). Therefore, we hypothesized that B. fragilis ZY-312 might promote an increase in the weight of rats through glucose metabolism. However, the exact mechanism needs to be explored through further study. Although ZY-312 may have some effect on neonatal rats, our previous studies of ZY-312 proved it to be safe and nontoxic (50).

## MATERIALS AND METHODS

### Bacterial strains and culture conditions.

Bacteroides fragilis strain ZY-312 (ZY-312) was provided by Zhiyi Biological Technology Co., Ltd., Guangzhou, China. The strain was cultured in Trypticase soy broth (TSB; Oxoid, Basingstoke, United Kingdom) with 5% fetal bovine serum (FBS; PAN-Biotech, Aidenbach, Germany) at 37°C for 18 h in an anaerobic incubator. Cronobacter sakazakii strain 29544 was purchased from the American Type Culture Collection (ATCC; Manassas, VA, USA) and grown overnight in Luria-Bertani (LB) or brain heart infusion (BHI) broth at 37°C. Human HT-29 intestinal epithelial cells were obtained from the American Type Culture Collection (ATCC, Manassas, VA, USA) and were cultured in RPMI 1640 medium with 10% FBS, 50 mg/ml penicillin G, and 100 mg/ml streptomycin. Human Caco-2 epithelial colorectal adenocarcinoma cells were purchased from the Shanghai Institute of Cell Biology (Shanghai, China) and cultured in RPMI 1640 medium with 20% heat-inactivated FBS, streptomycin (100 mg/ml), and penicillin G (50 mg/ml). All cells were grown at 37°C in a 5% CO_2_ incubator.

### Invasion assay.

Three different invasion assay procedures (competition, exclusion, and replacement) were performed as previously described (51). Briefly, HT-29 cells were seeded at 1 × 10^5^ cells per well in a 24-well tissue culture plate. Cell monolayers were washed with PBS and fresh RPMI 1640 medium with 10% FBS (without antibiotics). The C. sakazakii and ZY-312 cells were collected and suspended directly. HT-29 cells grown in 24-well tissue culture plates were infected with 1 × 10^7^ CFU of bacteria (with a multiplicity of infection [MOI] of 100) and incubated for 1.5 h as a control. For the competition assays, both 1 × 10^8^ CFU of ZY-312 and 1 × 10^7^ CFU of C. sakazakii were added to the cultures and incubated at 37°C for 3 h. For the replacement assays, cells were incubated with 1 × 10^7^ CFU of C. sakazakii at 37°C for 1.5 h and then 1 × 10^8^ CFU of ZY-312 was added to the cultures. For the exclusion assays, the cells were preincubated at 37°C with 1 × 10^8^ CFU of ZY-312 for 1.5 h and then 1 × 10^7^ CFU of C. sakazakii was added for 1.5 h, followed by incubation for 1.5 h (Fig. 1A). Monolayers were washed and incubated for a further 1.5 h with fresh medium containing gentamicin (100 g/ml). The cells were then washed and treated with 0.25% Triton X-100 for 8 min. Intracellular C. sakazakii cells were then enumerated by plating the treated cells on LB agar in duplicate. The results were expressed as relative levels of invasion compared to the parental C. sakazakii strain.

### Flow cytometric analysis.

HT-29 cells were incubated with C. sakazakii (MOI = 100) and ZY-312 (MOI = 1,000) for 3 h. Concurrently, cells were preincubated with ZY-312 for 3 h and then incubated with C. sakazakii for 3 h to assess the preventative effects. Cells were then harvested, washed, and resuspended in 500 μl of binding buffer containing 5 μl FITC-annexin V and 5 μl propidium iodide (PI) (Keygen, Nanjing, China). The cells were subjected to gentle vortexing and incubated for 15 min at 25°C in the dark. The degree of cell apoptosis was assessed by flow cytometry (BD Biosciences, CA, USA), based on the manufacturer’s instructions.

### Transepithelial electrical resistance (TEER) measurements.

Caco-2 cells were grown in Transwell inserts (Corning, NY, USA) for at least 21 days to form tight junctions. Cells were pretreated with 1 × 10^8^ CFU of ZY-312 for 3 h. C. sakazakii was then added to the upper chamber of the Transwell, followed by incubation. Transepithelial electrical resistance (TEER) across the Transwell filter was measured before and after C. sakazakii infection at 3 h, 6 h, and 18 h using a Millicell electrical resistance apparatus (World Precision Instruments, FL, USA).

### Periodic acid-Schiff (PAS) staining.

HT-29 cells were grown in 24-well plates and pretreated with bacteria. Cells were washed and fixed in 4% paraformaldehyde (PFA) at room temperature for 20 min. Staining was performed according to the instructions of the manufacturer (Solarbio Science & Technology Co., Ltd., Beijing, China) and viewed under a light microscope (Motic BA210).

### Immunofluorescence analysis.

Caco-2 monolayers or HT-29 cells were pretreated with bacteria. Cells were fixed in 4% PFA for 10 min at room temperature and then blocked in 5% normal goat serum–PBS for 1 h at room temperature. Subsequently, Caco-2 monolayers were probed with rabbit anti-ZO-1 antibody (1:200) and HT-29 cells were probed with rabbit anti-MUC2 antibody (1:100) for 12 h at 4°C. The cells were then incubated with Alexa Fluor 568-coupled goat anti-rabbit secondary antibody (Invitrogen, Carlsbad, CA, USA) at room temperature in the dark for 1 h. The membranes were mounted with Fluoroshield with DAPI (4′,6-diamidino-2-phenylindole) (Sigma-Aldrich, St. Louis, MO, USA) and examined under a fluorescence microscope (Nikon Eclipse TE2000-U).

### C. sakazakii-induced NEC in a neonatal rat model.

Specific-pathogen-free Sprague-Dawley neonatal rats were obtained from the Animal Experimental Center of the Southern Medical University (Guangzhou, China). NEC was induced in the neonatal rat model by C. sakazakii using a previously described protocol (52). A total of 40 rats were randomly allocated into four different groups: control, C. sakazakii, ZY-312, and prevention (pretreated with ZY-312 before exposure to C. sakazakii). The neonatal rats were breast-fed for 3 days after birth. Rats in the ZY-312 and prevention groups were fed with a 0.2 ml formula containing 1 × 10^9^ CFU of ZY-312 once per day. On day 3, the rats were fed with a 0.2 ml clean formula twice and exposed thrice to a hypoxia exposure regime with 5% O_2_ and 95% N_2_. Rats in the C. sakazakii and prevention groups were challenged with C. sakazakii (1 × 10^9^ CFU/rat) once per day. Fecal samples (*n* = 3 in each group), blood, and intestinal tissues were obtained 2 days after C. sakazakii infection (Fig. 2A). All animal experiments were approved by the Ethics Committee of the Southern Medical University and performed strictly according to the institute’s guidelines for animal care.

### Fluorescein isothiocyanate (FITC)-dextran permeability assay.

Intestinal epithelial barrier function was measured *in vivo*, using a 4-kDa fluorescein isothiocyanate (FITC)-dextran probe (Sigma-Aldrich) in serum. Rats were fasted overnight, followed by orogastric gavage of FITC-dextran (100 μl; 88 mg/ml in sterile PBS). After 4 h, rats were briefly exposed to gaseous carbon dioxide and euthanized by cervical dislocation. Blood samples were obtained via cardiac puncture and placed on ice until use. Blood samples were centrifuged at 5,000 rpm (4°C; 20 min), and then sera were collected. The FITC-dextran concentrations in the sera were determined by fluorometry (34).

### Immunohistochemical staining and histopathological examination.

For immunohistochemical staining, 5-μm-thick paraffin-embedded sections were deparaffinized and antigen retrieved. Sections were blocked in 1% normal goat serum and incubated overnight at 4°C with antibodies specific for MUC2, ZO-1, IgA, and iNOS (Abcam, Cambridge, United Kingdom), followed by incubation with horseradish peroxidase (HRP)-coupled secondary antibodies with 50 mM Tris-HCl buffer (pH 7.4) containing DAB (3,3′-diaminobenzidine) and H_2_O_2_. The sections were lightly counterstained with hematoxylin, and for histopathological examination, tissues were fixed by immersion in 10% neutral formalin, embedded in paraffin, cut into sections, and stained with hematoxylin and eosin (H&E). NEC was graded microscopically by two expert pathologists who were blind to the experimental groups. The pathology score ranged from 0 to 3. Grade 0 corresponds to normal architecture and healthy-appearing villi. Grade 1 corresponds to some mild evidence of inflammation without rearrangement of villus architecture or inflammatory cell infiltrate. Grade 2 is consistent with experimental necrotizing enterocolitis and corresponds to evidence of disruption of normal villi, sloughing, and inflammatory cell infiltrate. Grade 3 is characterized by loss of villi and histological evidence of perforation (5).

### Western blotting.

After protein quantification with a bicinchoninic acid (BCA) protein assay kit, samples were subjected to SDS-PAGE and transferred to polyvinylidene difluoride (PVDF) membranes, which were incubated overnight at 4°C with the following primary antibodies: ZO-1 (Abcam, MA, USA), occludin (Abcam, MA, USA), caspase-1 (Abcam, MA, USA), ASC (Abcam, MA, USA), NLRP3 (Abcam, MA, USA), IL-1β (Proteintech, Wuhan, China), GSDMD (Proteintech, Wuhan, China), caspase-3 (Affinity Biosciences, OH, USA), Bax (Affinity Biosciences, OH, USA), Bcl-2 (Affinity Biosciences, OH, USA), and β-actin (Affinity Biosciences, OH, USA). Protein bands were detected on a ChemiDoc-It system (Tanon, Shanghai, China) using an ECL kit (Bio-Rad, CA, USA). Protein levels were determined by the use of ImageJ. The density of each band was normalized to its respective loading control (β-actin). Each test was performed in three experiments with different sample batches.

### Microbial composition analysis.

Total genomic DNA from the samples was extracted using cetyltrimethylammonium bromide (CTAB)/SDS. DNA concentration and purity were assessed using 1% agarose gels. DNA was diluted to 1 ng/μl with sterile water. The V4 region of the 16S rRNA gene was amplified with 515F and 806R primers. In total, 12 samples were sequenced on an Illumina HiSeq 2500 platform (Illumina, CA, USA) provided by Novogene (Beijing, China). Paired-end reads were merged using FLASH (fast length adjustment of short reads), and sequence analysis was performed using UPARSE. Sequences with ≥97% similarity were assigned to the same operational taxonomic units (OTUs). The diversity and compositions of the bacterial communities were determined by estimating β diversity, according to Novogene’s recommendations (53).

### Statistical analysis.

All statistical analyses were performed using SPSS statistical software version 21.0 (IBM, NY, USA). Statistical differences between experimental groups were evaluated by Student’s *t* tests and one-way analysis of variance (ANOVA) with a Duncan multiple-range test or at least a significant difference test. All data were expressed as means ± standard deviations (SD). *P* values of ≤0.05 were considered statistically significant.

### Ethics statement.

All animal experiments were approved by Southern Medical University Animal Ethics Committee (protocol no. NFYY-2014-123), in accordance with relevant ethical principles and guidelines.

### Data availability.

Raw data corresponding to sequences of fecal samples were deposited into the NCBI’s Sequence Read Archive (SRA) under accession number PRJNA547866.
